# Interface State Density Prediction between an Insulator and a Semiconductor by Gaussian Process Regression Models for a Modified Process

**DOI:** 10.1021/acsomega.3c02980

**Published:** 2023-07-18

**Authors:** Kanta Matsunaga, Takuto Harada, Shintaro Harada, Akinori Sato, Shota Terai, Mutsunori Uenuma, Tomoyuki Miyao, Yukiharu Uraoka

**Affiliations:** † Graduate School of Science and Technology, Nara Institute of Science and Technology, 8916-5 Takayama-cho, Ikoma 630-0192, Nara, Japan; ‡ Data Science Center, Nara Institute of Science and Technology, 8916-5 Takayama-cho, Ikoma 630-0192, Nara, Japan

## Abstract

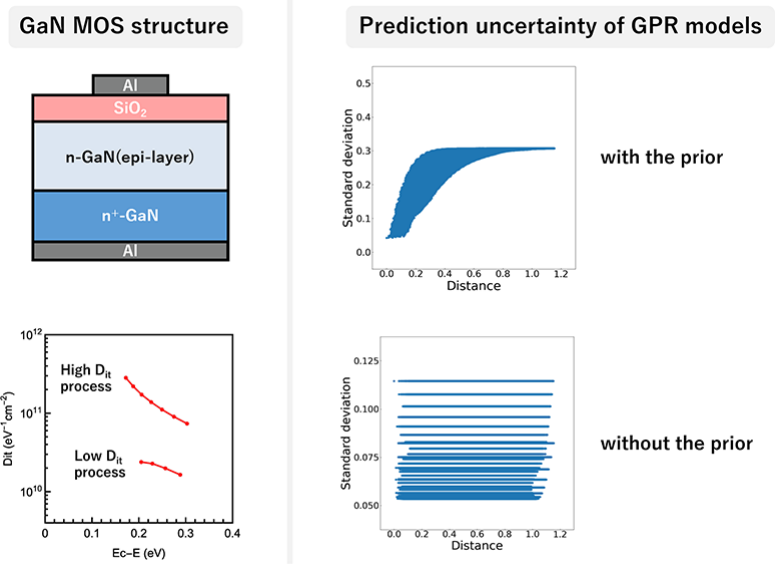

During data-driven process condition optimization on a laboratory scale, only a small-size data set is accessible and should be effectively utilized. On the other hand, during process development, new operations are frequently inserted or current operations are modified. These accessible data sets are somewhat related but not exactly the same type. In this study, we focus on the prediction of the quality of the interface between an insulator and GaN as a semiconductor for the potential application of GaN power semiconductor devices. The quality of the interface was represented as the interface state density, *D*_it_, and the inserted operation to the process was the ultraviolet (UV)/O_3_-gas treatment. Our retrospective evaluation of model-building approaches for *D*_it_ prediction from a process condition revealed that for the UV/O_3_-treated interfaces, data of interfaces without the treatment contributed to performance improvement. Such performance improvement was not observed when using a data set of Si as the semiconductor. As a modeling method, the automatic relevance vector-based Gaussian process regression with the prior distribution of the length-scale parameters exhibited a relatively high predictive performance and represented a reasonable uncertainty of prediction as reflected by the distance to the training data set. This feature is a prerequisite for a potential application of Bayesian optimization. Furthermore, hyperparameters in the prior distribution of the length-scales could be optimized by leave-one-out cross-validation.

## Introduction

Predictive machine learning (ML) models are frequently used in data-driven material discovery. Their usefulness has been proven with several material development projects, such as polymers, perovskites, and concrete mixtures.^1−3^ Although “big data” generated by high-throughput experimentation of material synthesis by combinatorial approaches can be sometimes obtained,^4^ at the laboratory scale, a limited number of samples, a small data set, can be usually accessible due to experimental cost and/or the difficulty of automation.

When utilizing a small data set for material discovery or process condition optimization, Bayesian optimization (BO) techniques can be used in combination with ML models to minimize the number of evaluations to find the optimal materials or process conditions.^5,6^ In BO, a set of candidate process conditions is proposed based on an acquisition function that evaluates the process conditions quantitatively. For successful BO, a surrogate ML model should be predictive and accurately represent the uncertainty of prediction based on which acquisition functions are designed. As the ML method for BO, Gaussian process regression (GPR) is frequently used.^7−9^ A GPR model predicts the distribution of the objective variable as a Gaussian distribution. The distribution reflects the similarity between the test process condition and the conditions used for training through the kernel function in the independent variable space. Thus, similar process conditions tend to show similar predicted property values. In practical applications, GPR models can be employed with the automatic relevance determination (ARD) technique^10^ to automatically reflect the importance of the variables on the length-scale parameters of the kernel function. However, when a data-set size is small, ARD might weigh only a few variables to be sensitive to the prediction and the rest of the variables are almost ignored (see the Effect of the Prior of Length-scales in GPR Section).

For predicting a material’s property from a set of process variables for fabricating the materials, ML models are normally built on all samples with all of the variables in the process. However, changing the process by inserting or removing an operation(s) makes the ML model construction procedure difficult. In general, there are two approaches to address this challenge. In one approach without data integration, samples from the original process are discarded or ignored completely, and only samples from the new process are used for model building. This might be effective and safe when the number of samples from the new process is large, and the measurement (synthesis) cost is low. In the other approach, data from the original process are merged with the ones in the new process to build an ML model. During process development for material design, the insertion/deletion of operations frequently occurs; thus, a way to integrate samples from different but similar processes is required; otherwise, many data points should be measured every time we change the process.

In the study of semiconductor devices, new operations are frequently inserted or current operations are modified. Because the quality of the gate dielectric and semiconductor interface is important, such as the metal-oxide-semiconductor (MOS) interface, various process technologies have been introduced, e.g., surface treatment of semiconductor substrates and heat treatment inserted in the middle of the process. While various process techniques have already been established for silicon semiconductors, process development is still required for wide-band-gap semiconductors. In this study, our objectives are GaN power semiconductor devices, which have attracted much attention in recent years, while many issues still remain at the MOS interface.^11,12^ One of the challenges is how to reduce the electrical interface defects measured by the interface state density, *D*_it_, which represents the quality of an interface. *D*_it_ is strongly affected by the surface condition of the GaN substrate and the deposition conditions of plasma-enhanced chemical vapor deposition (PECVD), which deposits SiO_2_. For improving the surface of GaN, previous research reported a significant reduction of *D*_it_ values by introducing the operation of UV/O_3_ treatment to the surface of GaN.^13^ As deposition conditions for PECVD, five process variables exist: temperature, pressure, radio frequency (RF) power, oxygen flow rate, and precursor material (TEOS) flow rate. Since UV/O_3_ treatment can be regarded as an inserted operation before the PECVD process on the GaN substrate, an effective way of data integration with and without UV/O_3_ treatment to make a highly predictive *D*_it_ model is important.

In this study, to identify effective model-building approaches for *D*_it_ prediction when the training data size was small and similar data sets were accessible, the *D*_it_ measurement data of interfaces between a semiconductor and the insulator of SiO_2_ with PECVD were prepared. The *D*_it_ values were measured by the Hi-Lo and conductance methods, and the semiconductors were Si and GaN. For some of the GaN samples, UV/O_3_ treatment was additionally applied. These samples were utilized for building predictive *D*_it_ models from the process variables. Employed ML methods were ARD-based GPR with and without the prior distribution of length-scale parameters. The ARD-based GPR were evaluated in terms of whether or not desired characteristics as a surrogate model for BO were achieved. These characteristics were the predictive ability of the model and the ability of reflecting distance to a training data set on the predicted distributions. A data integration strategy using a simple representation of one-hot encoding was tested using different but highly related process data sets of interfaces. We found that for predicting *D*_it_ for the UV/O_3_-treated interfaces of SiO_2_/GaN, the data set of the interfaces of SiO_2_/GaN without the treatment contributed to performance improvement. Such performance improvement was not observed when integrating a data set of SiO_2_/Si. Furthermore, ARD-based GPR models with the prior distribution of length-scales showed the desired characteristics as a surrogate model for BO.

## Materials and Methods

### Metal-Oxide-Semiconductor (MOS) Capacitor Fabrication

**Figure 1:**
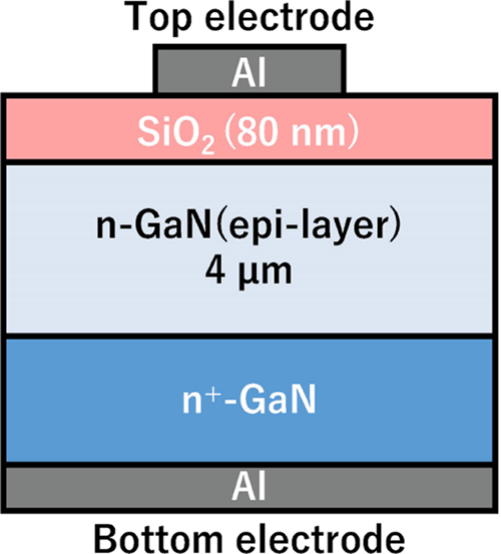
Schematic diagram of the fabricated MOS capacitor with the interface between SiO_2_ and *n*-GaN for measuring CV characteristics.

Si-doped GaN epitaxial layers (with a donor density of 5.0 × 10^16^ cm^–3^) of 4 μm thickness on an *n*-GaN(0001) substrate were used. The GaN surfaces were cleaned with buffered hydrofluoric acid (BHF) for 5 min and with hydrochloric acid (0.02 M) for 3 min. Then, the samples were rinsed with ultrapure water before the deposition of SiO_2_. After that, a thin film of SiO_2_ (thickness of ∼80 nm) was deposited by PECVD with TEOS and O_2_ gas. In the case of the surface-oxidized GaN sample (OxGaN), the GaN substrate was subjected to UV/O_3_ cleaning for 20 min at 100 °C before deposition of SiO_2_. SiO_2_/Si samples were fabricated using the same methods. After forming the Al electrodes, postmetallization annealing was carried out for 30 min at 400 °C under a N_2_ condition. The capacitance–voltage (CV) characteristics were evaluated, and the interface state density (*D*_it_) was calculated by the Hi-Lo method and conductance methods for SiO_2_/Si samples and SiO_2_/GaN (OxGaN) samples, respectively. Schematic diagram of the fabricated sample is provided in Figure 1.

### Data Sets for Machine Learning (ML)

*D*_it_ of SiO_2_ and a semiconductor interface was the objective variable: *y* in ML models. Two N-type substrates—GaN and Si—were employed for MOS capacitors. An operation with O_3_ gas was further applied to a few of them using GaN, leading to three types of interfaces: SiO_2_/Si, SiO_2_/GaN, and O_3_ gas-treated SiO_2_/GaN (OxGaN), corresponding to three data sets. Process variables in PECVD were independent variables (***x***) in ML models. These ML models can predict a *D*_it_ value from its process variable values. The ranges of *D*_it_ and the process variables for the three data sets are reported in Table 1. The number of samples for Si was 26, while that for OxGaN was 12. The *D*_it_ and the process variable values for the three data sets are provided in Table S1 in the Supporting Information. The values of the process variables were determined by our previous attempts to optimize the process variables targeting for lower *D*_it_ using BO approaches;^14^ thus, relatively diverse process variable values had been tested beforehand.

**Table 1:** Data-Set Profiles^a^

substrate	# samples	b*D*_it_ [eV^–1^cm^–2^]	Temp. [°C]	Press. [Pa]	RF power [W]	O_2_ flow [sccm]	TEOS flow [sccm]
GaN	24	[1.65 × 10^11^, 2.78 × 10^11^]	[154, 400]	[75, 250]	[30, 270]	[169, 999]	[1, 17.5]
Si	26	[2.61 × 10^10^, 7.53 × 10^11^]	[181, 400]	[72, 250]	[30, 266]	[169, 999]	[1, 17.6]
OxGaN	12	[1.40 × 10^10^, 1.31 × 10^11^]	[178, 400]	[75, 250]	[34, 270]	[169, 999]	[1, 17.5]

a For each target of semiconductorial material, the number of samples, the ranges of the process variables, and the objective variable are reported.

b *D*_it_ of the surface between the semiconductorial material and the insulator (SiO_2_).

### Gaussian Process Regression (GPR)

GPR is a regression algorithm based on the Gaussian process, where any finite collection of outputs of a function follows a multivariate Gaussian distribution. A trained GPR model can predict a Gaussian distribution of the objective variable for an arbitrary input ***x*** given a set of samples as a training data set. Thus, probabilistic operations, e.g., the expectation and the variance of the objective variable for ***x***, are naturally applied to GPR outputs. In general, GPR models are controlled by two parameters: the mean (*m*) and covariance functions (*k*) as shown in eq 1
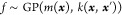
1

The mean and covariance functions give the mean vector and the covariance matrix of a multivariate Gaussian distribution for a collection of function outputs, respectively, governing the Gaussian process. Given a set of *n* data points *D* = {(***x***_1_,*y*_1_),(***x***_2_,*y*_2_),···,(***x***_*n*_,*y*_*n*_)}, the multivariate Gaussian distribution becomes
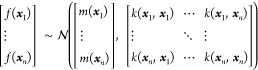
2where *m*(***x***_*i*_) represents the mean of *f*(***x***_*i*_) and *k*(***x***_*i*_,***x***_*j*_) represents the covariance between *f*(***x***_*i*_) and *f*(***x***_*j*_) for the *i-*th and *j*-th samples, respectively. In this study, *m*(***x***) takes a constant value. The covariance matrix in eq 2 is also called the kernel matrix (*K*). The kernel matrix determines the shape (smoothness) of the function based on **x** values. If two **x** points (***x***_1_ and ***x***_2_) take a high value of *k*(***x***_1_,***x***_2_), the corresponding output values (*f*(***x***_1_), *f*(***x***_2_)) become similar. In this study, the Matern52 kernel with ARD^10^ was used as the kernel function
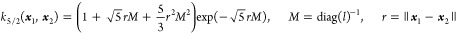
3where ***l*** = (*l*_1_,*l*_2_,···,*l*_*d*_) is a set of hyperparameters called length-scales, *d* is the dimension of ***x***, and ∥***x***_1_–***x***_2_∥ is the Euclidean distance between ***x***_1_ and ***x***_2_. ARD sets a length-scale parameter for each variable. A variable with a large length-scale value after optimization is regarded as unimportant to the model because the covariance of the model becomes almost independent of input values for the variable.^15^ In our study, the Matern52 kernel was further combined with a constant kernel to scale the output of the kernel
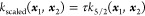
4

The derivation of the distribution of *y* for a new point is provided in Supporting Note S1.

#### Parameter Estimation for GPR

Parameters in GPR, such as the length-scales in eq 3 and the Gaussian noise σ^2^, are normally determined by maximizing the marginal likelihood for the observed data points. In this study, a prior distribution for each hyperparameter is introduced to avoid overfitting, in particular when data points are not many. To estimate the hyperparameters, maximum a posteriori (MAP) estimation was conducted based on the following equation
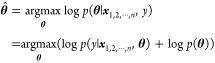
5

where ***θ*** = (*m*,***l***,τ,σ^2^) is a tuple of parameters, ***y*** = (*y*_1_,*y*_2_,···,*y*_*n*_). The logarithm of the marginal likelihood in eq 5 can be written when using the scaled kernel, *K*_scaled_
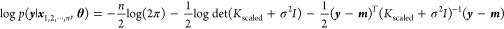
6

Assuming that the length-scales as well as the other parameters are independent, the prior distribution becomes as follows
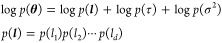
7

In this study, the prior distribution of each hyperparameter is given as a form of the γ distribution, γ(α,β), where α is the shape parameter and β is the inverse scale parameter (rate parameter). Each length-scale parameter takes the same γ distribution as prior. The logarithm of the prior (eq 7) can be represented as follows
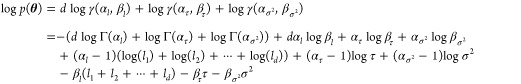
8where Γ represents the γ function and (α_*l*_,β_*l*_), (α_τ_,β_τ_), and (α_σ^2^_,β_σ^2^_) are the shape and inverse scale parameters for the length-scale, the scale parameter, and the Gaussian noise, respectively.

In this study, α_*l*_ = 3.0, β_*l*_ = 6.0, α_τ_ = 2.0, β_τ_ = 0.15, α_σ^2^_ = 1.1, and β_σ^2^_ = 0.05 were used for each prior hyperparameter, if not specified. The probability density functions for the length-scale, scale parameter, and noise are provided in Figure S1. These values were the default setting in the BOTorch library,^16^ which also showed relatively stable performance for the data sets in this study (see the Predictive Performance of ML Models Section).

### Software and Implementation

GPR models with/without prior distributions were implemented in an in-house Python script with the help of GPyTorch (version 1.9.0) and BOTorch modules.^17^

### ML Modeling Algorithms for Comparison

#### Random Forest Regressor

The random forest (RF) algorithm is an ensemble learning approach based on bagging.^18^ A set of bootstrap samples is prepared for each decision tree, and each tree is trained solely on the set with random features. The prediction by an RF regressor (RFR) is the average of the outputs of all trees. In this study, the scikit-learn implementation of RF was used: RandomForestRegressor (version 1.1.2). The hyperparameters of RF models were optimized by using the GridSearchCV module of scikit-learn with a set of parameters, provided in Table S2.

#### Multi-task Neural Networks

A simple multi-task neural network (multi-task NN) was used as a control. Multi-task NN can be trained on a data set mixed with multiple objective variables. In this study, the multiple objective variables correspond to *D*_it_ for the three substrates: GaN, Si, and OxGaN. The multi-task NN architecture is provided in Figure S2, which consisted of a sequence of input, hidden, and output layers. During the model training, the number of epochs was controlled to reach the best predicted *D*_it_ value for OxGaN as shown in Table S3.

### Evaluation Metrics

For evaluating *D*_it_ prediction models, the coefficient of determination (*R*^2^), the root-mean-square error (RMSE), and the mean absolute error (MAE) were used. These values are calculated by following the equations
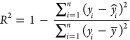
9
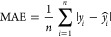
10
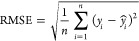
11

where *n* is the number of data, *ŷ*_*i*_ is the predicted value for the *i*-th data, and *y̅* is the average of the observed data.

### Data Preprocess

Before making ML models, independent variables (***x***) were scaled based on the theoretical maximum and minimum values for each variable. The logarithm form of *D*_it_ (log* D*_it_) was used followed by the range scaling using the maximum and minimum values of training data. The logarithm transformation allowed us to compare a wide range of possible *D*_it_ values, with different orders of magnitude. For comparison, we also made the GPR models using untransformed (original) *D*_it_ values.

## Results and Discussion

### Study Design

There were two objectives in this study for predicting *D*_it_ values from a limited number of samples: revealing an effective data integration strategy for ML models (1) and proposing the best modeling method that can be further utilized for BO (2). The limitation of the study is that all data were collected from a single laboratory with a well-established protocol and a small number of samples (12 for evaluation), meaning that experimental errors were expected to be small, and the derived conclusions can be supported when using a small data set.

**Figure 2:**
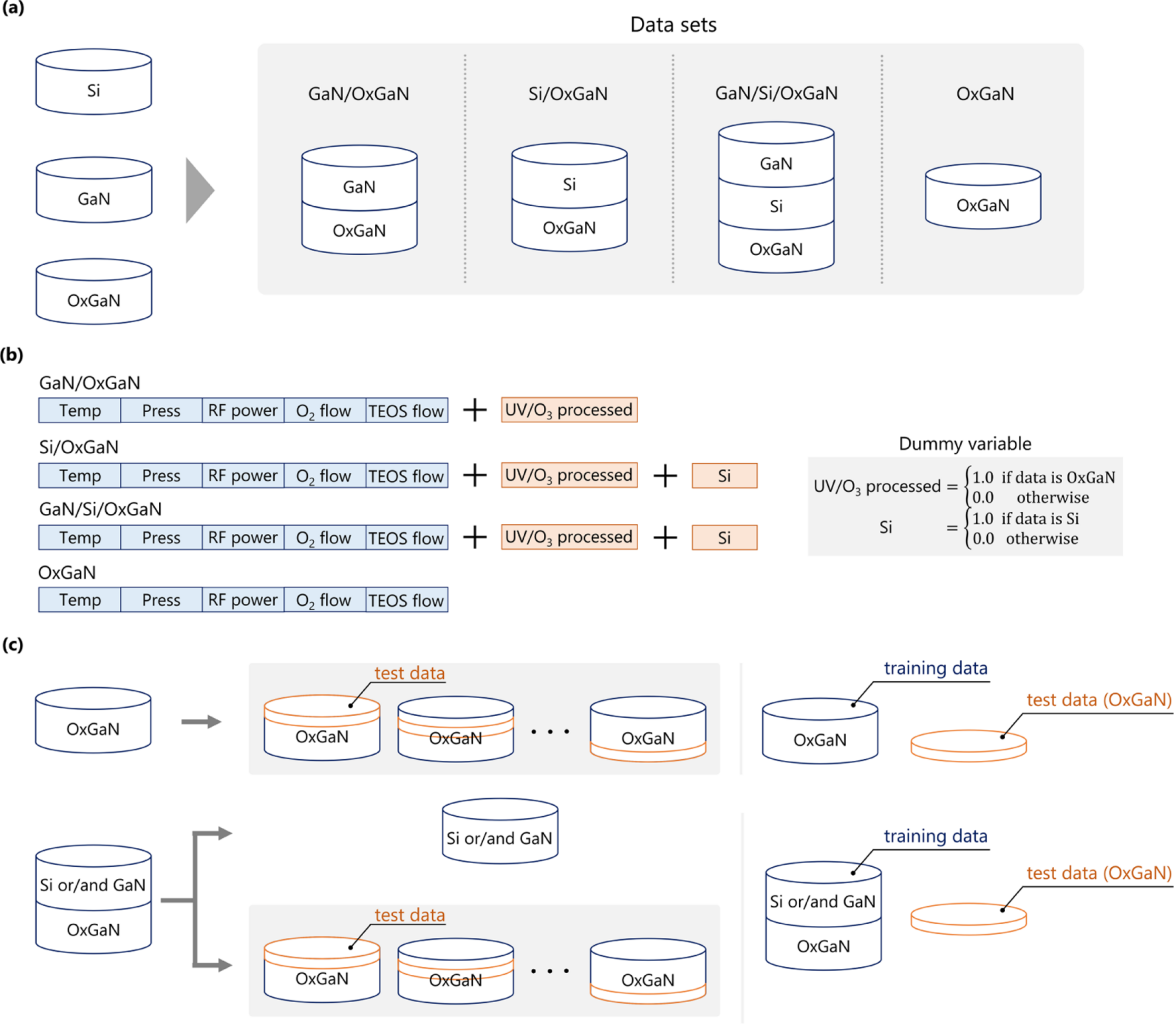
Evaluation scheme. Four integrated data sets were prepared (a). The features used for model building for each data set are described (b). In addition to the process variables, one-hot encoding vectors for the UV/O_3_ process and for Si were introduced for Si/OxGaN and GaN/Si/OxGaN data sets. For the OxGaN data set, the standard leave-one-out prediction was conducted (c upper), while for the other data sets, the OxGaN part was applied to leave-one-out splitting (c lower).

For the first objective, when targeting *D*_it_ prediction for OxGaN samples, four integrated data sets were prepared as training: GaN and OxGaN (GaN/OxGaN), Si and OxGaN (Si/OxGaN), GaN, Si, and OxGaN (GaN/Si/OxGaN), and only OxGaN. In ordinary ML approaches, training data sets show the same distribution as test data sets: using only OxGaN samples for both training and test (Figure 2a). However, different but somehow related samples might contribute to increasing the predictive performance of the model, i.e., OxGaN and GaN. This hypothesis was systematically tested in this study. To represent which type of interface is measured for *D*_it_, one-hot encoding of interface type was introduced (Figure 2b). A sample that was treated with O_3_ gas has a value 1 for the UV/O_3_-processed variable; otherwise, it is 0. Similar labeling was used for Si-based interfaces. To evaluate the performance of prediction models, leave-one-out cross-validation (LooCV) was conducted due to the relatively small number of OxGaN samples. The metrics of evaluations were *R*^2^, RMSE, and MAE. For a fair comparison with various ML models, GPR, RFR, and multi-task NN were used as regression models. For all ML models, a sample was represented by the five process variables and the two one-hot encodings (Figure 2b).

For the second objective, ARD-based GPR models with/without the prior distribution of length-scales were focused. Introducing the prior to the GPR modeling was expected to reduce the possibility of overfitting when a data set size was small. In addition to comparing the predictive ability among GPR models with/without the prior, the values of the optimized length-scales by maximizing marginal likelihood functions were scrutinized. Since BO is extensively utilized for material design when the number of samples is small, an appropriate quantification of the design space reflecting the training data distribution is important. In this respect, the predicted distributions for grid points in the design space were evaluated, which will become important information for further process variable optimization.

### Predictive Performance of ML Models

The LooCV-based performances of the prediction models for OxGaN samples are reported in Table 2. These models were combinations of the four integration strategies and the four modeling methods except for multi-task NN. Overall, the combinations of the GaN/OxGaN data set and GPR with/without the prior worked best. The *R*^2^ values for the two strategies were over 0.6, much higher than the other tested strategies. On the other hand, for the integration strategies involved in Si, the performances deteriorated drastically. For example, when using the Si/OxGaN data set, GPR with the prior had an *R*^2^ value of 0.27 and GPR without the prior had an *R*^2^ value of −0.46. These performance differences imply that there might be a relationship between the samples of GaN and those of O_3_-treated GaN, but not with Si. However, for the six pairs of samples having the same process variable values between GaN and OxGaN, the correlation coefficient of log* D*_it_ was −0.20, and one pair was found having the same process variable values between Si and OxGaN: 10.83 for Si and 10.64 for OxGaN. RFR showed the best performance when only the OxGaN data set was used, although the *R*^2^ values were much smaller than those with GPR models for the GaN/OxGaN data set. The hyperparameters of RFR were optimized by the inner grid search CV. In RFR models, when changing the number of folds in the CV, unstable prediction performances were observed except for the data set of OxGaN (Figure S3). This could explain the low predictive ability of RFR models when using the integrated data set. There is discrepancy between GPR with and without the prior approach. Without using the prior, prediction performances were unstable except for the GaN/OxGaN data set (Si/OxGaN: −0.46 in *R*^2^, OxGaN: −0.36). However, while using the prior, such performance deterioration was not observed (Si/OxGaN:0.27 in *R*^2^, OxGaN:0.29). Thus, introducing the prior had some merit in GPR models in this study. Multi-task NN showed a relatively good prediction performance (*R*^2^: 0.50), but it was lower than GPR with the prior and the GaN/OxGaN data set.

**Table 2:** Predictive Performance for OxGaN Samples^a^

	GPR with the prior			GPR without the prior			RFR			Multi-task NN		
data set	MAE	RMSE	*R*^2^	MAE	RMSE	*R*^2^	MAE	RMSE	*R*^2^	MAE	RMSE	*R*^2^
GaN/OxGaN	0.14	0.18	**0.67**	0.15	0.19	**0.63**	0.24	0.27	0.22			
Si/OxGaN	0.22	0.26	0.27	0.28	0.37	–0.46	0.23	0.26	0.28			
GaN/Si/OxGaN	0.23	0.28	0.19	0.62	1.1	–11.35	0.24	0.27	0.24	0.19	0.22	**0.50**
OxGaN	0.21	0.26	0.29	0.29	0.36	–0.36	0.23	0.25	**0.35**			

a For the four training data sets, looCV-based MAE, RMSE, and *R*^2^ are reported for the OxGaN samples. Four modeling methods—GPR w/wo the prior, RFR, and multi-task NN—were tested. For each modeling method, the highest *R*^2^ value is in bold.

The effect of the logarithm transformation of the objective variable on the model’s predictive ability was also investigated. As shown in Table S4, GPR with the prior without the log-transform overall performed poorly. On the other hand, without the prior distribution in GPR, the predictive performance of the model was almost as good as that of the model built on the log-transformed variable. For fairly comparing the evaluation metrics for the models with/without the logarithm transformation when the model was GPR without the prior and the data set was GaN/OxGaN, the predicted values (logarithm scale) were retransformed to the original scale. Although the model was trained for the logarithm-scaled variables, the metric values for the model using the logarithm-transformed variable were slightly better (MAE: 1.52 × 10^10^, RMSE: 1.99 × 10^10^, *R*^2^:0.66) than those for the model using the untransformed variable.

### Effect of the Prior of Length-scales in GPR

To further reveal the difference between GPR models with and without the priors, the optimized length-scale parameters (ARD) were calculated using all of the data in each training data set (Table 3). A large length-scale value for a variable indicates that the variable is unimportant for the GPR prediction. Without the prior when the data set was OxGaN, the only effective variable became RF power. This might be a result of the model overfitted to a small data size (12 samples). On the other hand, introducing the prior avoided relying only on a single variable. Rather the weights of variables were equally distributed among the process variables. For the GaN/OxGaN, the difference in length-scale parameters between GPR with and without the prior became smaller.

**Table 3:** Length-Scale Parameter Values in ARD

data set	prior	Temp.	Press.	RF power	O_2_ flow	TEOS flow	UV/O_3_ processed
GaN/OxGaN	yes	0.15	0.54	0.52	0.55	0.63	0.28
	no	0.14	0.97	0.70	1.2	2.7	0.18
OxGaN	yes	0.18	0.38	0.41	0.36	0.49	
	no	2.1 × 10^4^	7.6 × 10^3^	3.3	1.9 × 10^4^	4.9 × 10^3^	

To compare the model stability between GPR with and without the prior, the convergence of parameters by the maximization of marginal likelihood was tested by shuffling a training data set 10 times and building 10 GPR models for each OxGaN sample. In theory, the 10 models should predict the same distribution for each test sample. Using GPR with the prior, the average of the standard deviations for the training data sets was 9.0 × 10^–5^, while without the prior, the average was 9.5 × 10^–2^, meaning that the predicted distributions by GPR without the prior differed based on the order of training samples (Table S5).

**Figure 3:**
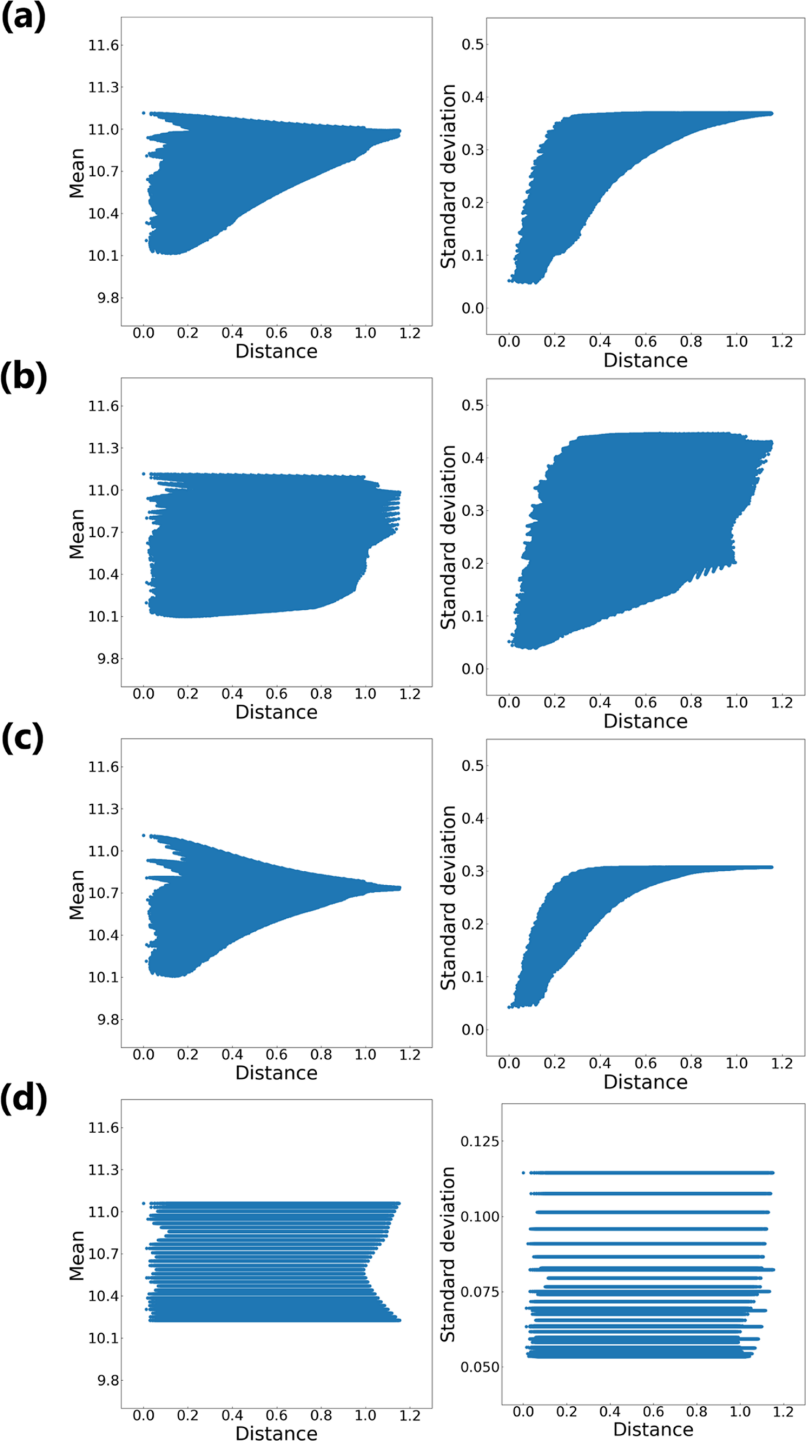
Predicted values for grid points in the experimental condition space. For each grid of exhaustive experimental conditions, predicted properties (mean and standard deviation) of *D*_it_ (*y* axis) are plotted against the distance to the training data set (*x* axis). The distance metric is the minimum Euclidean distance to training data points. The property values are predicted by GPR with the prior (a) and GPR without the prior (b) using GaN/OxGaN, and GPR with the prior (c) and GPR without the prior (d) using OxGaN.

To further investigate characteristics of the distribution of predicted *D*_it_ derived by GPR models with the optimized parameters, 24.3 million grid points in the process variable spaces were evaluated, which were the exhaustive combination of 30 equally split points for all of the process variables (30^5^). For the UV/O_3_-processed variable, a value of 1 was set when using the GaN/OxGaN data set. Figure 3 shows the predicted mean and standard deviation of *D*_it_ by GPR models for the grid points. Horizonal axes in Figure 3 are the minimum Euclidean distance to the training data points: a distance to the training data set. With the prior, the predicted mean and standard deviation values gradually converged as the distance to the training data set increased (Figure 3a,c). In particular, the average of the standard deviation values became larger as the distance increased. Most acquisition functions in BO depend solely on the predicted distributions. These characteristics of the GPR model with the prior seemed reasonable for BO. On the other hand, without the prior, the predicted mean and standard deviation values from GPR models did not converge as the distance increased (Figure 3b,d). In particular, the GPR model without the prior, which was trained on the OxGaN data set, predicted the same mean and standard deviation values irrespective of the distance. This was a consequence of the fact that the length-scale for only RF power had a smaller value in comparison to the other process variables (Table 3). When conducting BO with this model, the process variable values except for RF power will be determined almost at random.

### Hyperparameters of Length-scales Prior

#### Robustness of Hyperparameter Values on the Predictive Ability

**Figure 4:**
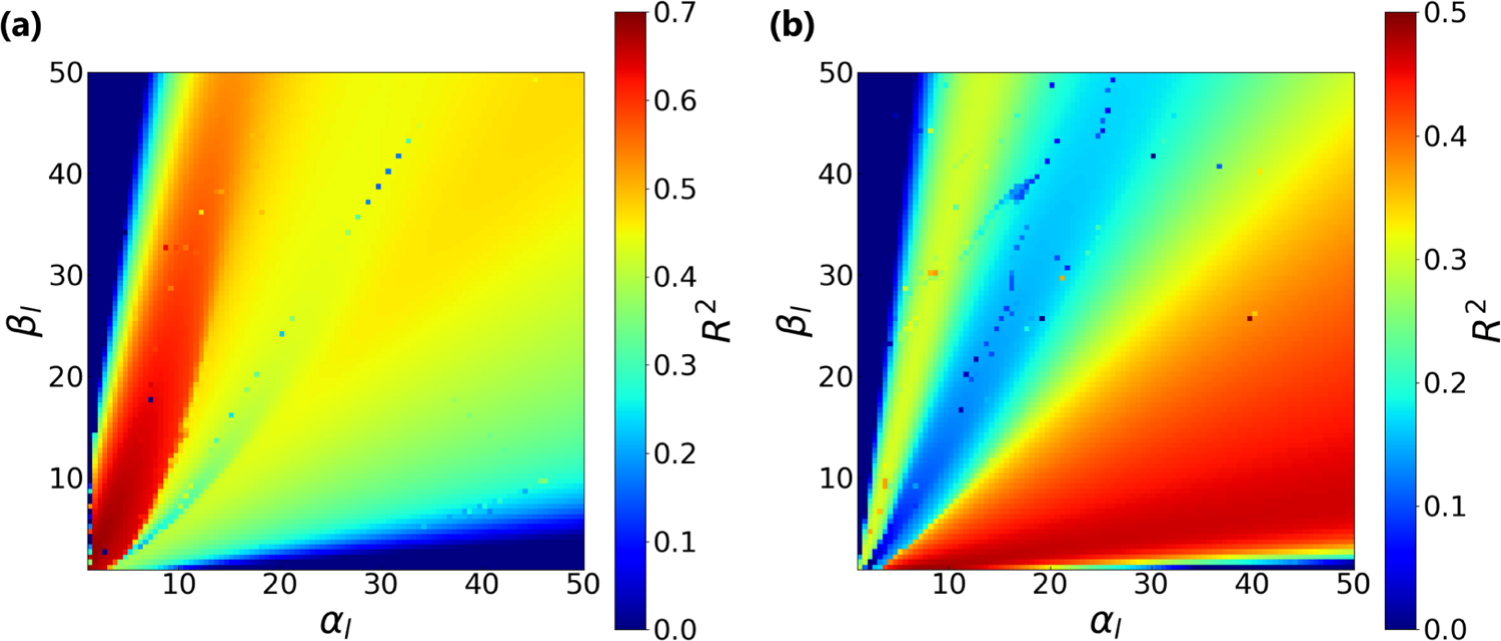
Effect of α_*l*_ and β_*l*_ on predictive performance. This figure shows the results of changing the α_*l*_ and β_*l*_ (α_*l*_,β_*l*_∈[1.0,1.5,···,50.0]) of the γ distribution, which is the prior distribution of the length-scale. There were some combinations of α_*l*_ and β_*l*_ having negative *R*^2^, but the range of color was assumed to be greater than 0. The property values are predicted by GPR with the prior using (a) GaN/OxGaN and (b) OxGaN.

Analyses in the previous sections revealed that setting a prior distribution to length-scales contributed to the higher predictive ability of the models and avoided predicting the same distributions for different test points. The prior for the length-scales was a γ distribution with parameters α_*l*_ and β_*l*_. The predictive ability of GPR models with the prior was systematically evaluated for exhaustive combinations of α_*l*_ and β_*l*_ from 1.0 to 50 with an interval of 0.5. Figure 4 reports LooCV-based *R*^2^ surfaces for the two data sets: GaN/OxGaN (Figure 4a) and OxGaN (Figure 4b). The top 10 performing combinations of α_*l*_ and β_*l*_ are also reported in Table 4. Obviously, extreme values for the parameters did not perform well for both data sets. On these two surfaces in Figure 4, combinations of the well-performed parameters differed: for GaN/OxGaN, α_*l*_ and β_*l*_ were similar values, while for OxGaN, larger α_*l*_ than β_*l*_ worked better. Since the γ distribution with α and β has a mean value of α/β, restricting each length-scale in a proper range of values might be key to building a better predictive model. In fact, for the top 10 performing models in Table 4, the average (the standard deviation) values of the optimized length-scales were Temp.: 0.14 (5.4 × 10^–3^), Press.: 0.61 (6.2 × 10^–2^), RF power: 0.55 (3.2 × 10^–2^), O_2_ flow: 0.61 (6.7 × 10^–2^), TEOS flow: 0.76 (0.15), and UV/O3 processed: 0.21 (8.3 × 10^–2^) when using the GaN/OxGaN data set, and Temp.: 6.7 (2.1), Press.: 6.8 (2.1), RF power: 5.2 (1.6), O_2_ flow: 6.6 (2.1), and TEOS flow: 7.0 (2.2) when using the OxGaN data set. The whole length-scale values after optimization are reported in Table S6. Although the scale of length-scales seemed restricted by the prior mean, the optimized length-scale values among the process variables varied. For the GaN/OxGaN data set, the length-scale of the Temp. variable was the smallest, while that of the TEOS flow was the largest (Table S6).

**Table 4:** Top Ten Performing Hyperparameter Combinations for Length-Scales

data set	α_*l*_	β_*l*_	*R*^2^	data set	α_*l*_	β_*l*_	*R*^2^
GaN/OxGaN	1.5	2.5	0.6836	OxGaN	39.5	25.5	0.4986
	1.0	1.0	0.6829		8.0	1.0	0.4867
	1.5	2.0	0.6825		7.5	1.0	0.4862
	1.5	3.0	0.6821		8.5	1.0	0.4861
	2.0	3.5	0.6816		9.0	1.0	0.4853
	2.0	3.0	0.6814		7.0	1.0	0.4851
	2.0	2.5	0.6801		9.5	1.0	0.4837
	2.0	4.0	0.6799		6.5	1.0	0.4833
	1.5	3.5	0.6794		10.0	1.0	0.4816
	2.5	4.0	0.6785		6.0	1.0	0.4804

When using a γ distribution relatively close to the unformal distribution (Figure S4), i.e., small α_*l*_ and β_*l*_ values, the predictive performances for the GaN/OxGaN data set showed good performance (*R*^2^: 0.68 for (α_*l*_,β_*l*_): (1.0, 1.0), 0.66 for (1.0, 0.1), and 0.66 for (1.0, 0.01)). On the other hand, for the OxGaN data set, too small values for β_*l*_ led to performance deterioration as *R*^2^: 0.29 for (α_*l*_,β_*l*_): (1.0, 1.0), −0.14 for (1.0, 0.1), and −0.17 for (1.0, 0.01).

### Optimization of Hyperparameters for the Length-scales Prior

Although the combination of the GaN/OxGaN data set and the ARD-based GPR with the prior was found to be suitable in terms of predictive ability (Table 2) and showed preferable characteristic for BO, reflecting the distance from a training data set on the predictive distributions (Table 3), these GPR models had the prior hyperparameter values predetermined by the BOTorch module as α_*l*_ = 3.0, β_*l*_ = 6.0. The important question was how to optimize these hyperparameters solely using a training data set. Thus, we conducted double CV or nested CV to optimize the hyperparameters.^19^ As the inner CV loop, LooCV was employed to determine the best hyperparameter values based on RMSE. The exhaustive combinations of α_*l*_ and β_*l*_ from 1.0 to 10 with an interval of 1.0 were searched in this process. Table 5 shows the predictive performance for LooCV when using the optimized combination of α_*l*_ and β_*l*_ for each OxGaN sample as a test. For each test sample, the fitting to the training samples inside the inner CV and the optimized combination of α_*l*_ and β_*l*_ are reported in Table S7. Furthermore, the predicted *D*_it_ value for each test sample by GPR with the prior with optimized α_*l*_ and β_*l*_ is provided in Table S8. The double CV-based validation for GPR with the prior also reached almost the same performance as using predetermined hyperparameter values in Table 2. Thus, hyperparameter values of α_*l*_ and β_*l*_ could be determined solely based on a training data set. However, when using only the OxGaN data set, fitting to the training data set sometimes failed possibly due to the small data-set size.

**Table 5:** LooCV Performance of GPR with the Optimized α_*l*_ and β_*l*_ Values

data set	MAE	RMSE	*R*^2^
GaN/OxGaN	0.16	0.19	0.62
OxGaN	0.25	0.27	0.24

## Conclusions

For data-driven material discovery or process optimization, an efficient ML model-building scheme is required when the data-set size is small and when introducing additional operations in the process. In this study, three types of interfaces fabricated by PECVD were prepared to evaluate their characteristics: SiO_2_/Si, SiO_2_/GaN, and UV/O_3_-treated SiO_2_/GaN. For each of the interfaces, a *D*_it_ value was measured and used as the objective variable value in ML models. We have shown that for predicting *D*_it_ values for the interfaces of UV/O_3_-treated SiO_2_/GaN, integrating SiO_2_/GaN samples into the target data set positively contributed to model predictive ability, but not SiO_2_/Si samples. Furthermore, as a surrogate model for BO, ARD-based GPR models with the prior of length-scales showed higher predictive ability, where the predicted distributions were reflected by distances to the training data set. The predictive performance by the GPR with the prior distribution models varied from different hyperparameters of the prior distribution yet could be optimized by LooCV using only a training data set.

## Supporting Information Available

Brief explanation on the predicted distribution of the output for a new point in GPR (Note S1); *D*_it_ and the process variables (Table S1); hyperparameters of RFR (Table S2); predictive performance for OxGaN by the multi-task NN models trained with different epochs (Table S3); predictive performance for OxGaN by GPR without the logarithm transformation of the objective variable (Table S4); stability of the GPR models (Table S5); length-scale values in the ARD-based GPR models using the top 10 performed combinations of α*_l_* and β*_l_* in the prior (Table S6); optimized α_*l*_ and β_*l*_ values in the double CV trial (Table S7); predicted *D*_it_ value for each test sample using optimized and in the double α*_l_* and β*_l_* CV trial (Table S8); probability density functions (PDFs) for length-scale, scale parameter and noise (Figure S1); multi-task neural network architecture (Figure S2); effect of fold sizes on the predictive ability of RFR models (Figure S3); probability density functions (PDFs) with small α and β values (Figure S4) (PDF)
