# Supplementary material for: Interface State Density Prediction between an Insulator and a Semiconductor by Gaussian Process Regression Models for a Modified Process
Source: ACS Omega. 2023 Jul 18;8(30):27458–66. doi: 10.1021/acsomega.3c02980 (PMC10398861; doi:10.1021/acsomega.3c02980)

Supporting Information

Interface State Density Prediction between Insulator and Semiconductor by Gaussian Process Regression Models for a Modified Process

Kanta Matsunaga[1], Takuto Harada[1], Shintaro Harada[1], Akinori Sato[1], Shota Terai[1], Mutsunori Uenuma[1,2]* Tomoyuki Miyao[2,1]* Yukiharu Uraoka[1,2]

[1]Graduate School of Science and Technology, Nara Institute of Science and Technology, 8916-5 Takayama-cho, Ikoma, Nara, 630-0192, Japan.

[2]Data Science Center, Nara Institute of Science and Technology, 8916-5 Takayama-cho, Ikoma, Nara, 630-0192, Japan.

*Corresponding authors:

Mutsunori Uenuma: uenuma@ms.naist.jp

Tomoyuki Miyao: miyao@dsc.naist.jp

Supporting Note S1. Predicting the distribution of the output for a new point.

The multivariate Gaussian distribution becomes

$$\begin{bmatrix} f(\boldsymbol{x}_1) \\ \vdots \\ f(\boldsymbol{x}_n) \end{bmatrix} \sim \mathcal{N}\left(\begin{bmatrix} m(\boldsymbol{x}_1) \\ \vdots \\ m(\boldsymbol{x}_n) \end{bmatrix}, \begin{bmatrix} k(\boldsymbol{x}_1,\boldsymbol{x}_1) & \dots & k(\boldsymbol{x}_1,\boldsymbol{x}_n) \\ \vdots & \ddots & \vdots \\ k(\boldsymbol{x}_n,\boldsymbol{x}_1) & \dots & k(\boldsymbol{x}_n,\boldsymbol{x}_n) \end{bmatrix}\right), (S1)$$

where $m(\boldsymbol{x}_i)$ represents the mean of $f(\boldsymbol{x}_i)$ and $k(\boldsymbol{x}_i,\boldsymbol{x}_j)$ represents the covariance between $f(\boldsymbol{x}_i)$ and $f(\boldsymbol{x}_j)$ for the i-th and j-th samples, respectively.

Assuming that values of an objective variable $\{y_1, y_2, \cdots, y_n\}$ independently follow the Gaussian distribution with a variance of σ^2 centered on $f(\boldsymbol{x}_k)$, we have

$$\boldsymbol{y} \sim \mathcal{N}\left(\begin{bmatrix} f(\boldsymbol{x}_1) \\ \vdots \\ f(\boldsymbol{x}_n) \end{bmatrix}, \sigma^2 I\right). (S2)$$

And the marginal distribution of $\boldsymbol{y}$ over $\boldsymbol{f}$ given $\boldsymbol{X}$ is provided as

$$p(\boldsymbol{y}|\boldsymbol{X}) = \int p(\boldsymbol{y}|\boldsymbol{f},\boldsymbol{X})p(\boldsymbol{f}|\boldsymbol{X})d\boldsymbol{f}. (S3)$$

Because both components in **eq. S3** are Gaussian functions, the marginal distribution is also a Gaussian with the mean vector of $\boldsymbol{m}$ and the covariance matrix of $K + \sigma^2 I$.

When predicting the distribution of the output for a new point of $\boldsymbol{x}^*$, the noise-free distribution $f(\boldsymbol{x}^*)$ or f^* can be analytically derived as a Gaussian distribution conditioned on the observed data points: $D = \{(\boldsymbol{x}_1, y_1), (\boldsymbol{x}_2, y_2), \dots, (\boldsymbol{x}_n, y_n)\}$ from the joint distribution of $\boldsymbol{y}$ and f^*:

$$p(f^*|\boldsymbol{x}^*, D) = \mathcal{N}(m(\boldsymbol{x}^*) + \boldsymbol{k}_*^T(K_{scaled} + \sigma^2 I)^{-1}(\boldsymbol{y} - \boldsymbol{m}), k_{**} - \boldsymbol{k}_*^T(K_{scaled} + \sigma^2 I)^{-1}\boldsymbol{k}_*),$$

$$\boldsymbol{k}_* = \left(k(\boldsymbol{x}^*, \boldsymbol{x}_1), k(\boldsymbol{x}^*, \boldsymbol{x}_2), \cdots, k(\boldsymbol{x}^*, \boldsymbol{x}_n)\right)^T,$$

$$k_{**} = k(\boldsymbol{x}^*, \boldsymbol{x}^*), (S4)$$

where $\boldsymbol{m}$ is the mean vector in **eq. S1**, and K_{scaled} is the covariance matrix of the scaled kernel values.

Table S1. D_{it} and the process variables.

Substrate	D_{it} [$eV^{-1}cm^{-2}$]	Temp. [℃]	Press. [Pa]	RF power [W]	O_2 flow [sccm]	TEOS flow [sccm]
GaN	3.83E+11	300	80	150	300	3
	3.32E+11	368	75	180	643	17.5
	6.18E+10	200	238	240	903	12.8
	4.11E+10	178	222	239	858	12.9
	4.55E+10	177	239	236	838	14.4
	1.65E+11	154	201	253	882	15.2
	1.26E+11	155	212	206	828	12.9
	1.69E+11	154	224	253	736	11.8
	5.69E+10	185	202	235	732	14.4
	4.53E+10	181	225	240	880	12.5
	4.37E+11	243	125	60	406	2.6
	9.19E+10	306	175	120	169	7.6
	2.78E+11	400	250	270	999	1
	4.15E+10	200	250	237	953	13.5
	4.72E+10	184	222	200	945	14.5
	1.53E+11	312	250	228	873	10.8
	1.08E+11	304	250	249	925	14.2
	9.33E+10	314	250	201	901	14. 7
	5.42E+10	303	250	198	843	15.7
	5.26E+10	297	250	202	941	16.0
	4.06E+10	184	250	213	999	11.6
	1.39E+11	299	197	159	192	12.5
	1.10E+11	385	160	95	550	13
	5.03E+10	400	250	30	849	1
Si	4.15E+10	300	80	75	300	7
	5.72E+10	300	80	150	300	1
	1.49E+11	300	80	75	300	3
	2.87E+11	300	80	225	300	3
	2.39E+11	300	80	150	300	6
	4.25E+11	300	80	150	300	9
	6.7E+10	300	80	150	300	3
	4.51E+11	244	175	266	880	12.9
	5.31E+11	181	125	63	643	8.1
	1.18E+11	306	75	131	169	3.4
	2.58E+11	368	225	199	406	17.6
	5.43E+10	300	80	145	303	1.4
	4.85E+10	309	78	142	335	1.1
	5.65E+10	289	74	143	329	1
	9.73E+10	400	250	30	999	1
	4.04E+10	304	86	150	366	1
	7.53E+11	304	79	44	299	8.6
	4.72E+10	300	94	160	366	1
	4.73E+10	301	87	160	367	1
	3.43E+10	303	82	162	419	1

	2.61E+10	302	82	167	402	1
	3.77E+10	304	81	165	437	1
	3.46E+10	304	94	155	380	1
	2.97E+10	308	94	173	407	1
	2.61E+10	302	72	171	394	1
	3.04E+10	311	94	156	385	1
OxGaN	4.37E+10	300	80	150	300	3
	8.66E+10	178	222	239	858	12.9
	6.48E+10	368	75	180	643	17.5
	1.31E+11	400	250	270	999	1
	1.44E+10	243	125	60	406	2.6
	2.08E+10	306	175	120	169	7.6
	1.61E+10	246	135	56	384	7
	3.59E+10	220	156	84	303	3
	3.03E+10	291	122	58	264	4.6
	4.45E+10	286	142	77	516	4.4
	1.40E+10	233	94	76	489	6
	1.68E+10	226	125	34	580	3.9

Table S2. Hyperparameters of RFR.

Parameters	Candidate
n_estimators	50,100,500,1000
max_depth	None,2,4,6,8,10
max_features	None, sqrt,log2
min_samples_leaf	1,2,3

Table S3. Predictive performance for OxGaN by the multi-task NN models trained with different epochs.

Epoch	MAE	RMSE	R^2
10	0.26	0.30	0.05
50	0.24	0.26	0.27
100	0.23	0.25	0.33
200	0.20	0.22	0.48
300	**0.19**	**0.22**	**0.50**
400	0.19	0.23	0.46
500	0.20	0.24	0.39
1000	0.23	0.28	0.17

Table S4. Predictive performance for OxGaN by GPR without the logarithm transformation of the objective variable.

Data set	GPR with the prior			GPR without the prior		
	MAE	RMSE	R^2	MAE	RMSE	R^2
GaN/OxGaN	2.35E+10	3.23E+10	0.08	1.63E+10	2.10E+10	0.62
Si/OxGaN	5.17E+10	6.57E+10	-2.74	4.01E+10	4.85E+10	-1.03
GaN/Si/OxGaN	5.29E+10	6.62E+10	-2.78	4.21E+10	4.83E+10	-1.02
OxGaN	1.90E+10	2.84E+10	0.30	2.60E+10	3.34E+10	0.04

Table S5. Stability of the GPR models. For each test sample, the average and the standard deviation of predicted log D_{it} values when using 10 shuffled training data sets (GaN/OxGaN) are reported.

OxGaN sample	GPR with prior		GPR without prior	
	Average log D_{it}	standard derivation	Average log D_{it}	standard derivation
1	10.63	3.9E-04	10.54	9.0E-02
2	10.97	7.6E-07	10.69	**0.32**
3	10.98	9.7E-05	10.85	**0.16**
4	10.98	3.1E-06	10.95	5.4E-02
5	10.27	1.6E-06	10.19	6.4E-04
6	10.76	8.7E-05	10.74	5.2E-02
7	10.23	9.9E-05	10.19	8.2E-05
8	10.34	5.1E-07	10.28	5.6E-04
9	10.57	1.7E-04	10.47	**0.15**
10	10.44	1.2E-04	10.34	**0.28**
11	10.27	5.8E-05	10.23	2.2E-03
12	10.33	5.7E-05	10.30	3.0E-02

Table S6. Length-scale values in the ARD-based GPR models using the top 10 performed combinations of α_l and β_l in the prior.

Data set	α_l, β_l	Temp.	Press.	RF power	O_2 flow	TEOS flow	UV/O_3 processed
GaN/OxGaN	1.5,2.5	0.14	0.60	0.54	0.61	0.76	0.19
	1.0,1.0	0.14	0.70	0.58	0.72	1.02	4.10E-05
	1.5,2.0	0.14	0.67	0.57	0.66	0.89	0.23
	1.5,3.0	0.14	0.55	0.52	0.56	0.68	0.17
	2.0,3.5	0.14	0.59	0.54	0.59	0.73	0.25
	2.0,3.0	0.14	0.63	0.57	0.64	0.80	0.26
	2.0,2.5	0.15	0.70	0.60	0.70	0.90	0.29
	2.0,4.0	0.14	0.55	0.52	0.56	0.66	0.23
	1.5,3.5	0.13	0.51	0.49	0.52	0.63	0.14
	2.5,4.0	0.15	0.62	0.57	0.52	0.49	0.29
OxGaN	39.5,25.5	1.52	1.53	1.49	1.52	1.53	-
	8.0,1.0	7.29	7.43	5.60	7.21	7.57	-
	7.5,1.0	6.80	6.94	5.16	6.72	7.07	-
	8.5,1.0	7.78	7.93	6.03	7.70	8.06	-
	9.0,1.0	8.27	8.42	6.47	8.18	8.56	-
	7.0,1.0	6.31	6.45	4.73	6.23	6.57	-
	9.5,1.0	8.75	8.90	6.92	8.65	9.07	-
	6.5,1.0	5.82	5.96	4.30	5.74	6.08	-
	10.0,1.0	9.24	9.39	7.35	9.15	9.55	-
	6.0,1.0	5.33	5.46	3.87	5.26	5.57	-

Table S7. Optimized α_l and β_l values in the double CV trial. For each test sample, the optimized hyperparameter values along with fitting to the training data are reported.

OxGaN test sample No.	GaN/OxGaN				OxGaN			
	α_l, β_l	MAE	RMSE	R^2	α_l, β_l	MAE	RMSE	R^2
1	2.0,1.0	0.015	0.023	1.00	9.0,1.0	0.14	0.15	0.63
2	4.0,3.0	0.017	0.024	1.00	9.0,1.0	0.14	0.15	0.54
3	3.0,3.0	0.014	0.021	1.00	9.0,1.0	0.14	0.15	0.59
4	2.0,1.0	0.016	0.023	1.00	2.0,4.0	0.0046	0.0050	1.00
5	4.0,3.0	0.018	0.025	1.00	8.0,1.0	0.13	0.14	0.61
6	2.0,1.0	0.016	0.025	1.00	10.0,1.0	0.11	0.13	0.75
7	3.0,1.0	0.017	0.024	1.00	8.0,1.0	0.14	0.15	0.58
8	4.0,3.0	0.017	0.025	1.00	2.0,3.0	0.0028	0.0035	1.00
9	2.0,2.0	0.013	0.020	1.00	6.0,1.0	0.12	0.14	0.71
10	4.0,3.0	0.018	0.026	1.00	5.0,1.0	0.11	0.13	0.78
11	3.0,2.0	0.016	0.024	1.00	6.0,1.0	0.12	0.13	0.69
12	2.0,2.0	0.013	0.020	1.00	7.0,1.0	0.14	0.15	0.57

Table S8. Predicted D_{it} value for each test sample using optimized α_l and β_l in the double CV trial. For each test sample, predicted and observed D_{it} values are reported.

OxGaN test sample No.	GaN/OxGaN				OxGaN			
	α_l, β_l	obs.	pred.	Absolute Error	α_l, β_l	obs.	pred.	Absolute Error
1	2.0,1.0	10.64	10.50	0.14	9.0,1.0	10.64	10.48	0.16
2	4.0,3.0	10.94	10.93	0.01	9.0,1.0	10.94	10.64	0.30
3	3.0,3.0	10.81	10.97	0.16	9.0,1.0	10.81	10.61	0.20
4	2.0,1.0	11.12	10.96	0.16	2.0,4.0	11.12	10.61	0.51
5	4.0,3.0	10.16	10.27	0.11	8.0,1.0	10.16	10.37	0.21
6	2.0,1.0	10.32	10.71	0.39	10.0,1.0	10.32	10.63	0.31
7	3.0,1.0	10.21	10.17	0.04	8.0,1.0	10.21	10.36	0.15
8	4.0,3.0	10.55	10.23	0.32	2.0,3.0	10.55	10.24	0.31
9	2.0,2.0	10.48	10.59	0.11	6.0,1.0	10.48	10.30	0.18
10	4.0,3.0	10.65	10.40	0.25	5.0,1.0	10.65	10.38	0.27
11	3.0,2.0	10.15	10.25	0.10	6.0,1.0	10.15	10.42	0.27
12	2.0,2.0	10.23	10.32	0.09	7.0,1.0	10.23	10.33	0.10

Figure S1. Probability density functions (PDFs) for length-scale, scale parameter and noise.

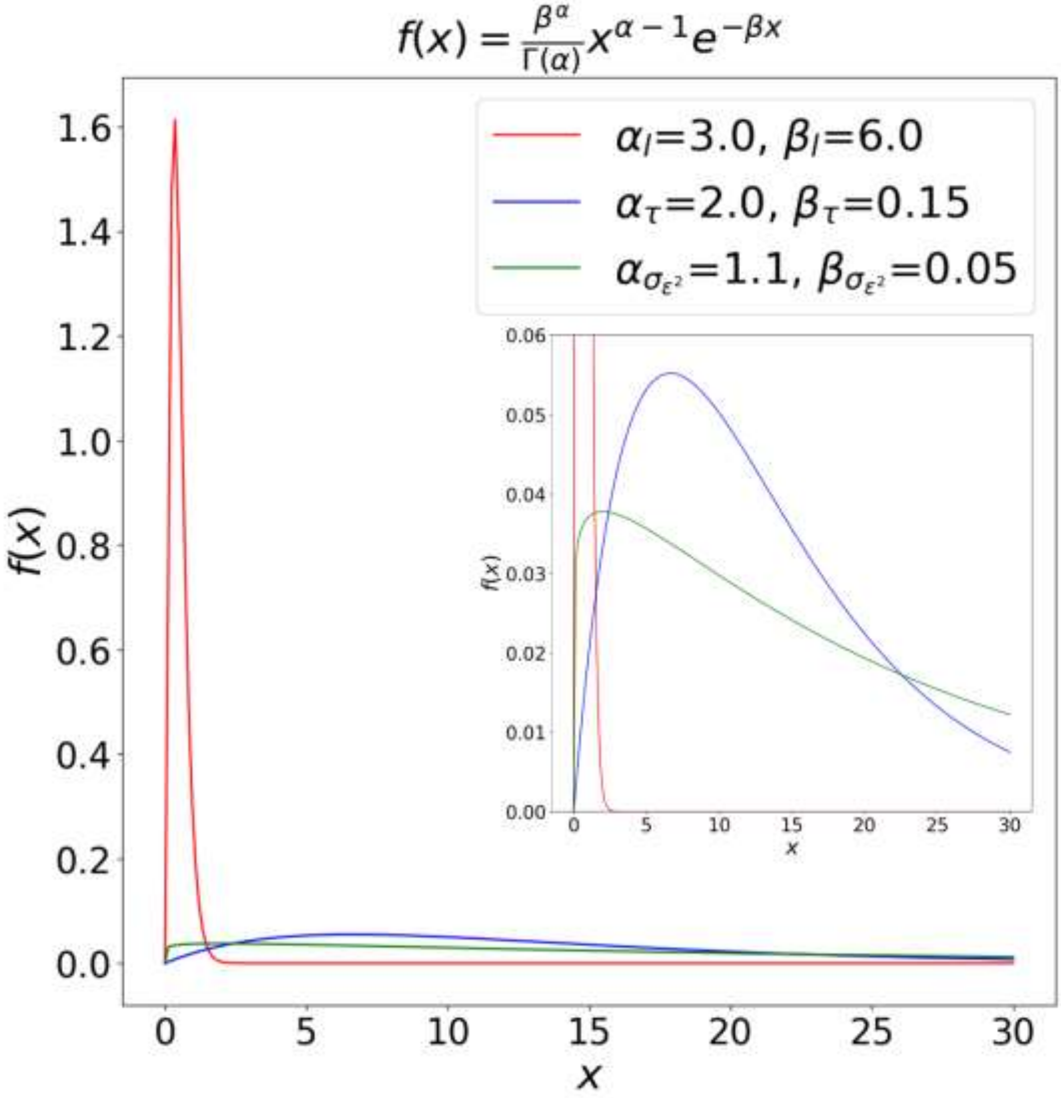

Figure S2. Multi-task neural network architecture. The number of nodes in the input layer was identical to the number of variables (seven), including the five deposition conditions and the two indicators for the processes. The number of nodes in hidden layer was 16. The outputs were log-transformed D_{it} values for the three processes. Rectified linear units (ReLU) was used as the activation function of the hidden layer. The loss function was the squared error function.

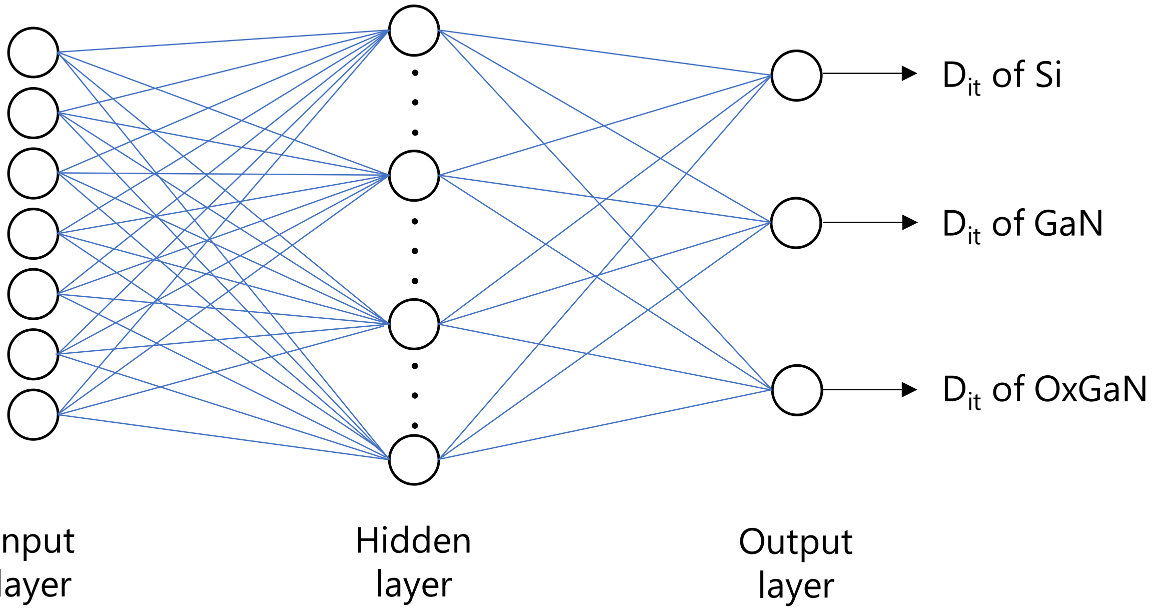

Figure S3. Effect of fold sizes on the predictive ability of RFR models. Lower numbers of folds resulted in unstable predictive ability by RFR models. The x-axis shows the fold number of inner CV for the hyperparameter optimization of RFR models, and the y-axis the predicted R^2 value for LOO-based OxGaN data set.

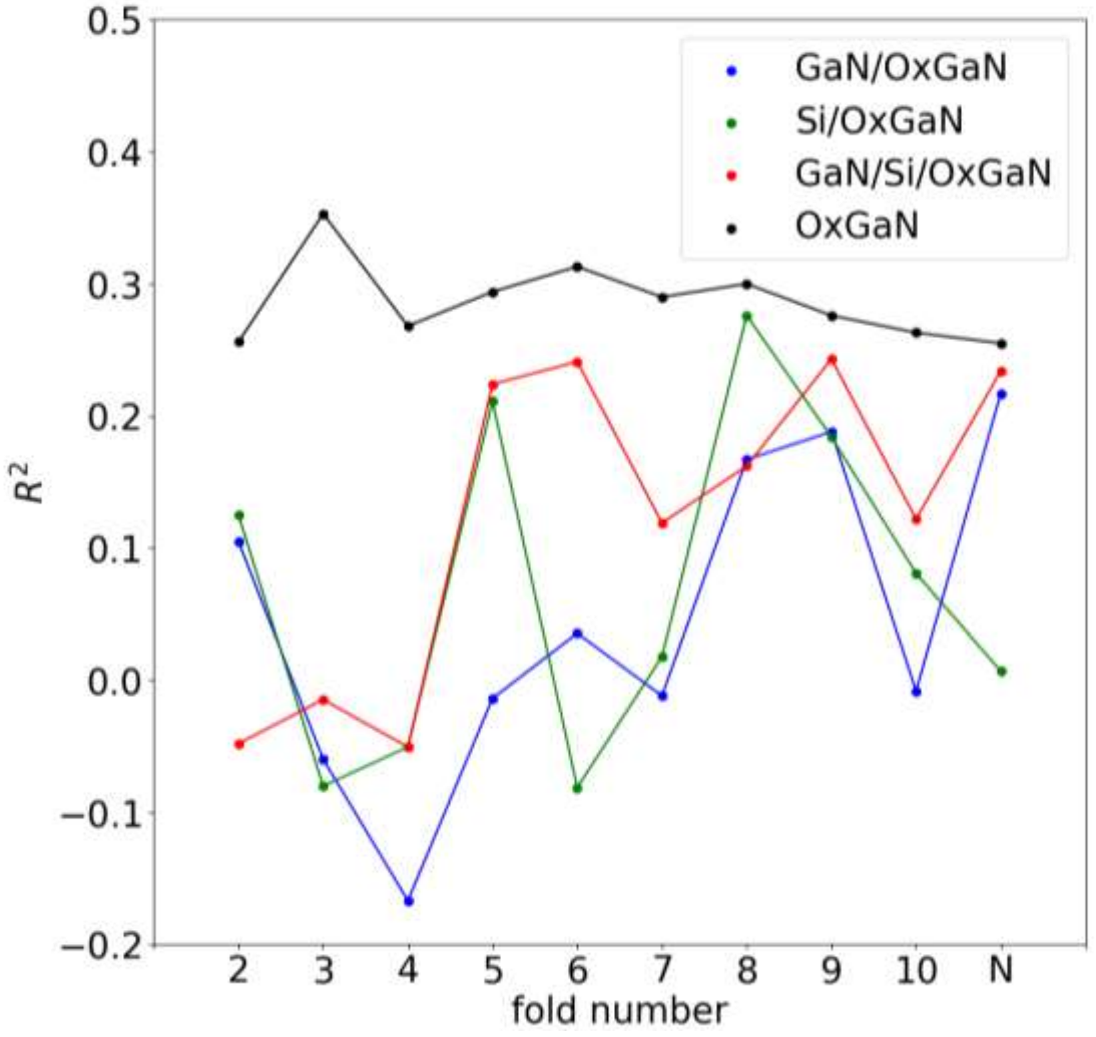

Figure S4. Probability density functions (PDFs) with small α and β values.

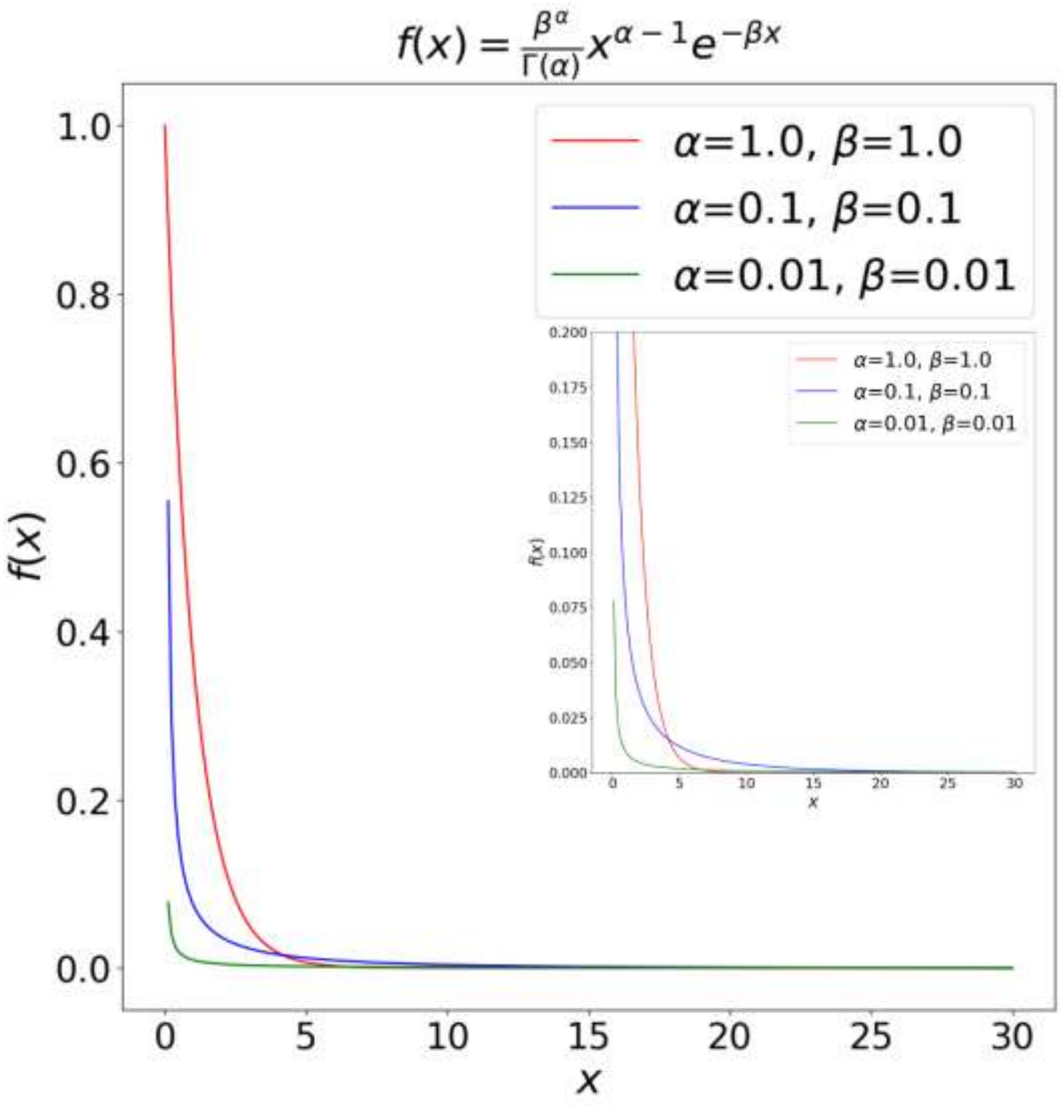

Supplement: Supplementary file 1 — ao3c02980_si_001.pdf [file ao3c02980_si_001.pdf]
